# Effects of Pressure Level and Time Treatment of High Hydrostatic Pressure (HHP) on Inulin Gelation and Properties of Obtained Hydrogels

**DOI:** 10.3390/foods10112514

**Published:** 2021-10-20

**Authors:** Anna Florowska, Tomasz Florowski, Barbara Sokołowska, Lech Adamczak, Iwona Szymańska

**Affiliations:** 1 Institute of Food Sciences, Warsaw University of Life Sciences-SGGW, 159c Nowoursynowska Street, 02-787 Warsaw, Poland; tomasz_florowski@sggw.edu.pl (T.F.); lech_adamczak@sggw.edu.pl (L.A.); iwona_szymanska@sggw.edu.pl (I.S.); 2 Department of Microbiology, Prof. Wacław Dąbrowski Institute of Agricultural and Food Biotechnology—State Research Institute, 36 Rakowiecka Street, 02-532 Warsaw, Poland; barbara.sokolowska@ibprs.pl; 3 Institute of High Pressure Physics of Polish Academy of Sciences, 29/37 Sokołowska Street, 01-142 Warsaw, Poland

**Keywords:** inulin hydrogels, high hydrostatic pressure, gelation, gel properties, gel stability

## Abstract

The aim of this study was the evaluation of the influence of different HHP levels (150 and 300 MPa) and time treatment (5, 10, 20 min) on the gelation and properties of hydrogels with different inulin concentration (15, 20, 25 g/100 g). High-pressure treatment, in tested ranges, induces inulin gels and allows obtaining gel structures even at a lowest tested inulin content (i.e., 15 g/100 g). Selecting the pressure parameters, it is possible to modify the characteristics of the created hydrogels. The use of higher pressure (i.e., 300 MPa) allows to increase the stability of the hydrogels and change their structure to more compressed, which results in higher yield stress, lower spreadability, harder and more adhesive structure. For example, increasing the inulin gelling induction pressure (concentration 20 g/100 g) from 150 to 300 MPa with a time treatment of 10 min resulted in an increase in yield stress from 38.1 to 711.7 Pa, spreadability force from 0.59 to 4.59 N, firmness from 0.11 to 1.46 N, and adhesiveness from −0.06 to −0.65 N. Extending the time treatment of HHP increases this effect, but mainly when higher pressure and a higher concentration of inulin are being used. For example, extension of time treatment at 300 MPa pressure from 5 to 20 min resulted in an increase in yield stress from 774.8 to 1273.8 Pa, spreadability force from 6.28 to 8.43 N, firmness from 1.87 to 2.98 N, and adhesiveness from −0.94 to −1.27 N. The obtained results indicate the possibility of using HHP to create inulin hydrogels tailored to the characteristics in a specific food product.

## 1. Introduction

Hydrogels are a group of polymeric materials of three dimensional cross-linked polymeric networks capable of absorbing and retaining a significant amount of aqueous solvents and biological fluids in their structures [1]. They are listed as “smart structures” whose tailor-made design gives them various functional features for use in the designing, synthesis and self-assembly of novel biomaterials and drug delivery systems. Hydrogels were used for the first time in the production of contact lenses. Since then, they have been widely used, among others in controlled drug delivery, agriculture, water purification and food technology [2]. Hydrogels can be obtained by physical cross-linking [3], chemical cross-linking [4], polymerization grafting [5], and radiation cross-linking [6]. Commonly in the food industry physical cross-linking is being used due to their relatively facile production and the advantage of not using the cross-linkers. Methods, described in the literature, to produce physically cross-linked hydrogels include: heating or cooling a polymer solution [7], ionic interactions [8], complex coacervation [9], hydrogen bonding [10], maturation or aggregation resulting from heat [11], and cryogelation [12]. Other methods that have recently gained popularity in the food industry include high hydrostatic pressure (HHP). HHP is being used to disorder biopolymers, due to the modifications of non-covalent intermolecular interactions resulting in inducing gelatinization [13]. The HHP processing on an industrial scale in food production is being used for improving the efficiency of food production, or as non-thermal pasteurization or blanching technique to inactivate microorganism in juices, fruits, vegetables, seafood, and eggs. The reason why HHP is gaining popularity is that changes in nutritional and sensorial properties of food are minimize during that treatment. There is a particular need in using HHP as gelation inducers because the pressure-induced gelatinization is considered significantly different from heat-induced gelatinization [14]. HHP treatment is known to make morphological and structural changes in polysaccharides, resulting in production food with novel texture and hydrogels with different properties compared to conventional methods. In general, HHP does not accelerate chemical reactions, but rather causes physical changes in molecules microscopically and/or macroscopically. Among others things, movements are limited while hydrogen bonds are broken and the molecules are packed together toward filling the gaps between them [15]. HHP is usually carried out under pressures ranging from 50 to 1000 MPa [16]. In the available literature the range of tested HHP levels on gelation of polysaccharides are between 100 and 600 MPa, as it is for starch [17,18], glucomannan [19] and carrgeenan [20]. The selection of process parameters depends on the type of polymer, pressure level, time of treatment and temperature. Those parameters can play an important role in determining the presence and degree of hydrogel formation as well as its properties.

Hydrogels used in food products are produced mostly from natural polysaccharides such as alginate, gelatine, cellulose, chitosan and starch [21]. Inulin, by its chemical composition and structure can, similarly to other polysaccharides, undergo physical or chemical crosslinking into tri-dimensional polymeric networks resulting in obtaining hydrogels [22]. The widespread use of inulin is due to its properties such as: being a prebiotic food ingredient with textural properties, non-toxic proved by its Generally Recognized as Safe (GRAS) status granted by the Food and Drug Administration (FDA), biocompatible, water soluble, biodegradable and relatively low cost polymer [23]. It is known that inulin can also form hydrogels after HHP (500 MPa) treatment [24]. However, it is not known whether lower pressure also allows them to induce gelation, and if so, what influence the pressure parameters (pressure level and time treatment) have on the characteristics of the gels formed. This could allow for the optimization of the food production process with inulin, where the use of high pressures at the level of 500 MPa is not required (obtaining a microbiological effect) or is not recommended due to unfavorable changes in the product. That is why the aim of this work was to evaluate the influence of different HHP levels (150 and 300 MPa) and time treatments (5, 10, 20 min) on the induction and properties of inulin hydrogels with different inulin concentration (15, 20, 25 g/100 g).

## 2. Materials and Methods

### 2.1. Materials

The material used in the experiment was inulin Orafti^®^ HPX (average degree of polymerization DP ≥ 23) purchased from BENEO GmbH (Mannheim, Germany).

#### Formation of Inulin Gels

Inulin hydrogels were induced by high hydrostatic pressure (HHP); firstly inulin (15, 20, 25 g/100 g) was suspended in distilled water (20 °C) using a magnetic stirrer (for approximately 5 min). Then the solution were pour into plastic cylindrical bottles (50 mL; high 0.075 m; ø 0.035 m) and exposed to pressure of 150 and 300 MPa at a temperature of 20 °C for 5, 10 and 20 min. Pressure build-up time was 100 s and the release time was 2–4 s. The tested pressurization times did not include the build-up and release times. The working volume of the treatment chamber was 0.95 L. Distilled water and polypropylene glycol in a ratio of 1 : 1 were used as the pressure-transmitting fluid. Samples were subjected to high pressure at the Institute of High Pressure Physics, The Polish Academy of Science, using U 4000/65 (Unipress, designed and produced by the Laboratory of High Pressure Equipment) apparatus. In the apparatus U4000/65 the pressure was generated by a hydraulically driven high-pressure pump (boxer-type) and was released by means of a hydraulically driven high-pressure value. The pressure limit of U4000/65 apparatus was 600 MPa and the temperature of HP chamber was controlled by water circulation through the wall of the chamber.

After pressurization samples were stored at refrigerated temperature (8 °C) for 24 h until the structure was build and the inulin gels were formed, than quality feature of induced gels were tested. 

Samples were prepared in three experimental separate replicates.

### 2.2. Methods

#### 2.2.1. Volumetric Gel Index (VGI)

For valuation of the of degree of hydrogels formation the VGI was used. VGI was measured in a plastic cylindrical bottles (50 mL) in which inulin solutions were exposed for pressure treatment. The volumetric gel index (VGI) was expressed as [25]:

$$VGI=\frac{VG}{VT}\times 100\;\left[\%\right]$$

where: *VG*—volume of formulated gel, *VT*—total volume of sample.

For hydrogel samples in which gel structure is not formed VGI is equal 0, and for the completely gelled samples VGI will equal 100%.

#### 2.2.2. Determination of Physical Stability

Physical stability has been demonstrated as a space and time related transmission profiles. The physical stability of inulin hydrogels was analyzed with LUMiSizer 6120-75 (L.U.M. GmbH, Berlin, Germany). During the analysis the following parameters were used for the measurement: wavelength 870 nm, volume 1.8 mL of dispersion; light factor: 1; 1500 rpm; experiment time, 15 h 10 min; interval time 210 s; temperature 20 °C. The instability index was calculated using the SepView 6.0; LUM (Berlin, Germany) software. It was quantified by dividing the clarification of the sample at a given separation time, by the maximum sample clarification. The instability index took values in the range 0 and 1, where 0 indicates a stable system and 1 an unstable system [26].

#### 2.2.3. Microstructure

The differences in hydrogels microstructure was analyzed using an electron scanning microscope (FEI Quanta 200 ESEM, Hillsboro, OR, USA) equipped with an energy dispersive spectrometer (EDS) and digital image recording. Hydrogels were previously freeze-dried and attached to a band made of carbon and covered with gold, and then microscopically examined. Specimens were observed at pressures of 100–133 Pa, under accelerating voltage of 25 or 30 kV. Analyses of images of the gel structure were done using MultiScan v.18.03 software (Computer Scanning System).

#### 2.2.4. Yield Stress 

Yield stress (Pa) of hydrogels through steady state viscometry was measured using a rheometer (DV3T, Brookfield, Middleboro, MA, USA) at 20 °C, equipped with a vane four knife spindles V74 with a torque range HA. The constant share rate was 0.1 s^−^^1^. The reported values represent the averages of six replicates. The values of yield stress were analyzed using the equipment software.

#### 2.2.5. Textural Properties

Selected texture parameters (firmness, adhesivness and spreadability) of hydrogels were measured using TA.XT Plus (Stable Microsystems, Surrey, UK) with a 5 kg load cell at 20 °C. The firmness (N) and adhesiveness (Ns) were measured with a cylindrical of 0.5-cm diameter (P/0.5R) probe penetrating the gel for 8 cm distance, the measurement speed was 1.0 mm/s, and trigger force was 0.01 N. The spreadability was measurement with a TTC Spreadability Rig. The test speed and distance were set to 3.0 mm/s and 20 mm, respectively. Force [N] was measured for the duration of the test, and spreadability [N*s] was equated to the area under the curve. The reported values in all textural parameters represent the averages of six replicates. The data were analyzed using the Exponent version 6.1.16.0 equipment software.

#### 2.2.6. Color Parameters 

The L*, a*, and b* color components of inulin hydrogels were analyzed at the surface of inulin gels, with a Minolta CR-200 colorimeter (Minolta, Japan; light source D65, a measuring head hole of 8 mm). In order to determine the color differences between inulin hydrogels prepared with different pressures and time treatment the total color difference ΔE was calculated according to formula [27]:

$$\Delta E=\sqrt{{\left(L_{HHP1}^{*}-L_{HHP2}^{*}\right)}^{2}+{\left(a_{HHP1}^{*}-a_{HHP2}^{*}\right)}^{2}+{\left(b_{HHP1}^{*}-b_{HHP2}^{*}\right)}^{2}}$$

where: $L_{HHP1}^{*},\;a_{HHP1}^{*},b_{HHP1}^{*}$ and $L_{HHP2}^{*},\;a_{HHP2}^{*},b_{HHP2}^{*}$ refers to the color parameters of compared hydrogels.

Depending on the ΔE values the color difference between the samples can be estimated as not noticeable for the observer (0 < ∆E < 1), only experienced observer can notice the difference (1 < ∆E < 2), unexperienced observer also notices the difference (2 < ∆E < 3.5), clear difference in color is noticed (3.5 < ∆E < 5) and observer notices two different colors (5 < ∆E) [27].

#### 2.2.7. Statistical Analysis

The obtained results were analyzed statistically using Statistica13.3 (TIBICO Software Inc., Tulsa, OK, USA). To determine the significance of differences between the average values of yield stress, firmness, adhesiveness, spreadability, color parameters of inulin hydrogels one-way Anova analysis of variance was used. Significant differences between different HHP treatments: pressure level, time treatment for particular inulin concentration, and differences between inulin concentrations for the same HHP treatment were verified using Tukey’s test at significant level α = 0.05. 

## 3. Results and Discussion

### 3.1. The Influence of HHP Level and Time Treatment on Formation and Stability of Inulin Hydrogels

Inulin suspensions with concentrations in the range 15–25 g/100 g were used to investigate gel formation under high pressure levels (150, 300 MPa) at different times treatment (5, 10, 20 min). On the basis of the conducted research, it was found that the use of even the lowest from the tested pressures (150 MPa) and lasting for a shorter time (5 min) allowed us to induce the gelation process and, as a result, to create a gel structure (VGI = 100 %, Table 1). This effect was found in all tested inulin concentrations, even the smallest, i.e., 15 g/100 g. According to the available literature data, this concentration was insufficient for other induction methods (thermally or with the use of shear forces) where the lowest concentration forming a gel structure (VGI = 100%) was 20 g/100 g [28,29,30]. This may indicate the potential possibility of using high pressures to create inulin gel structures in food products even in lower inulin concentration.

It is known from previously published results [24] that with a pressure of 500 MPa it is possible to induce a hydrogel structure, however, the present studies show that this structure can be obtained also at much lower pressures. Lower levels of pressure (e.g., 300 MPa) are being indicated in publications considering using HHP as potential shelf life prolonger in typical food application [31,32,33]. What is more, different properties of food product might be obtained using pressure in range between 100–300 MPa. For example, such an effect was found in the case of viscosity of mango pulp, which increased after HHP treatments at 100 or 200 MPa (20 °C/15 or 30 min), and was reduced after HHP treatments at 300 and 400 MPa (20 °C/15 or 30 min) [34].

**Figure 1:**
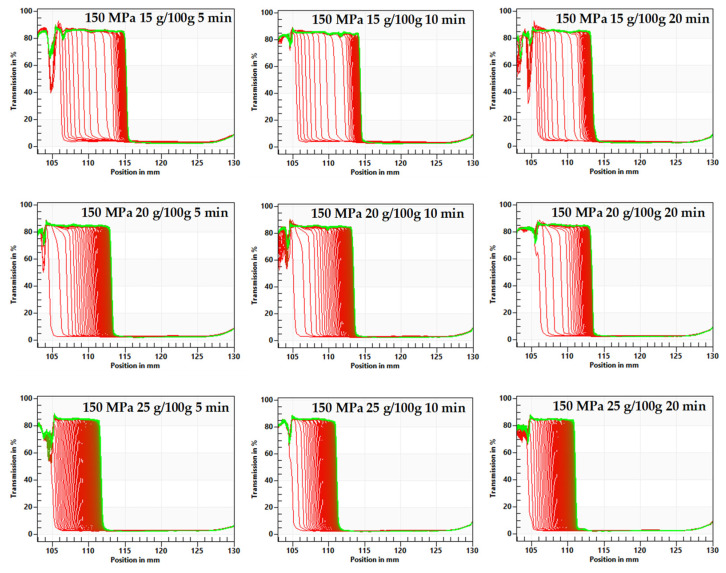
Influence of time treatment at pressure 150 MPa on inulin gel transmission profiles presented enabling LUMiSizer^®^ analysis.

**Figure 2:**
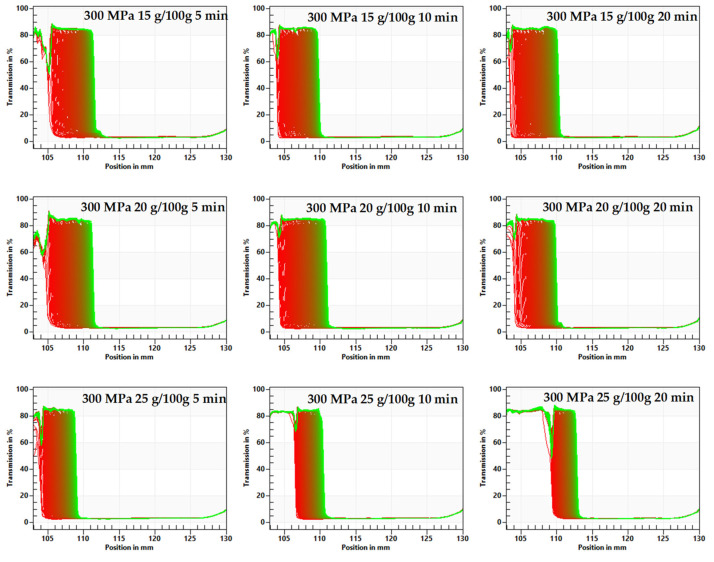
Influence of time treatment at pressure 300 MPa on inulin gel transmission profiles presented enabling LUMiSizer^®^ analysis.

The research showed that although at each tested concentration of inulin and all applied pressurization parameters, hydrogels were formed, they were characterized by different stability. The effect of pressurization parameters on hydrogels stability was examined with the multi-sample analytical centrifuge based on the STEP technology (space-time resolved extinction profiles). The transmission profiles of hydrogels showed high start transmissions over 80%. The evaluation of transmission profiles indicated particle movement toward the sedimentation process. Gels induced at lower pressures (150 MPa) were less stable than those induced at higher (300 MPa) pressures (Figure 1 and Figure 2 show space-time resolved extinction profiles).

**Figure 3:**
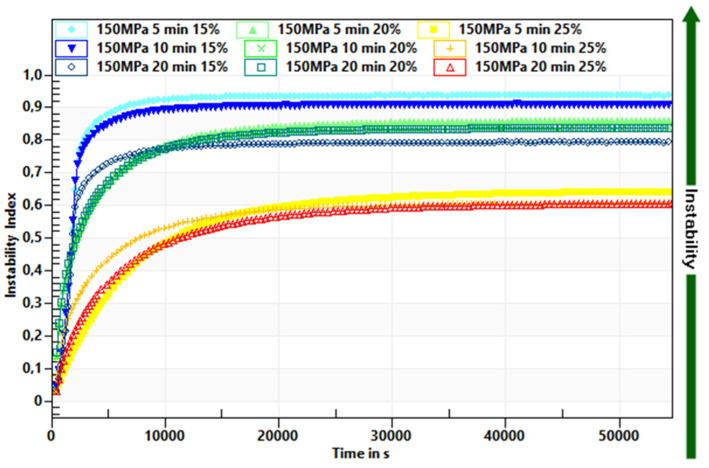
Influence of time treatment at pressure 150 MPa on inulin gel instability index.

**Figure 4:**
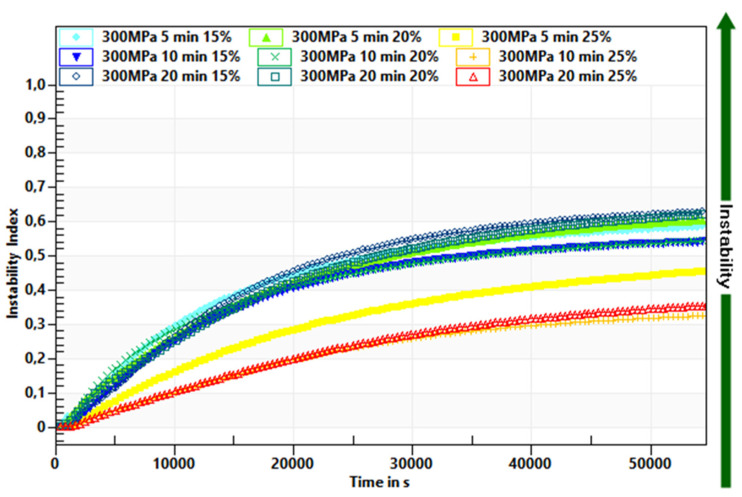
Influence of time treatment at pressure 300 MPa on inulin gel instability index.

A faster destabilization process was observed in hydrogels induced by lower pressure level and in shorter time treatment, as shown by decrease of the transmission profiles. The Instability Index values for hydrogels induced with pressure of 150 MPa were 0.6 and higher (Figure 3) whereas those induced with pressure of 300 MPa were below 0.6 (Figure 4). Additionally, it was observed that time of pressure treatment also had an impact on the stability of hydrogels. Extending the time (from 5 to 20 min) of holding the solutions at a conducted pressure resulted in the production of more stable hydrogels. Such trends were observed regardless of the inulin concentration, although it was found that gels obtained from solutions with higher inulin concentrations were more stable. The observed trends in the influence of pressure parameters were in accordance with the observations made for other polysaccharides—starch [35] and pectin [36].

### 3.2. The Influence of HHP Level and Time Treatment on Microstructure of Inulin Hydrogels

**Figure 5:**
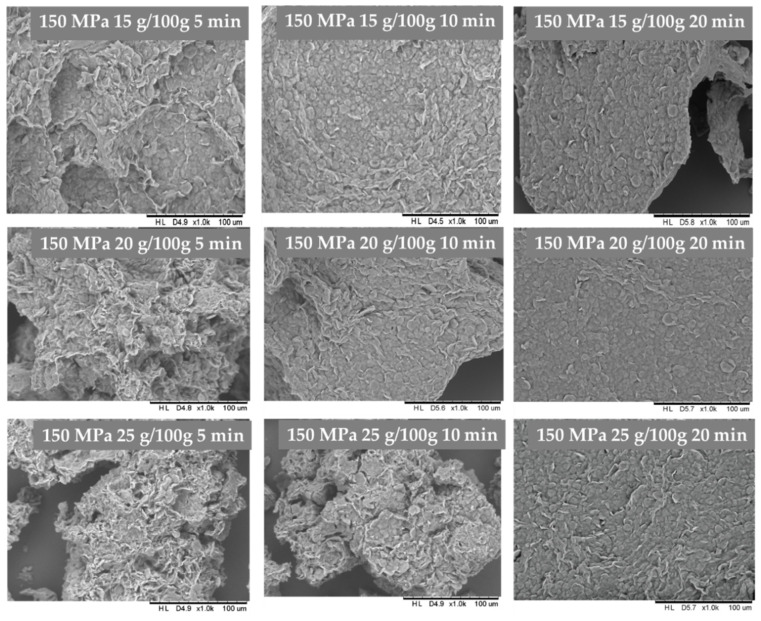
Influence of HHP time treatment under 150 MPa on inulin gel structure.

**Figure 6:**
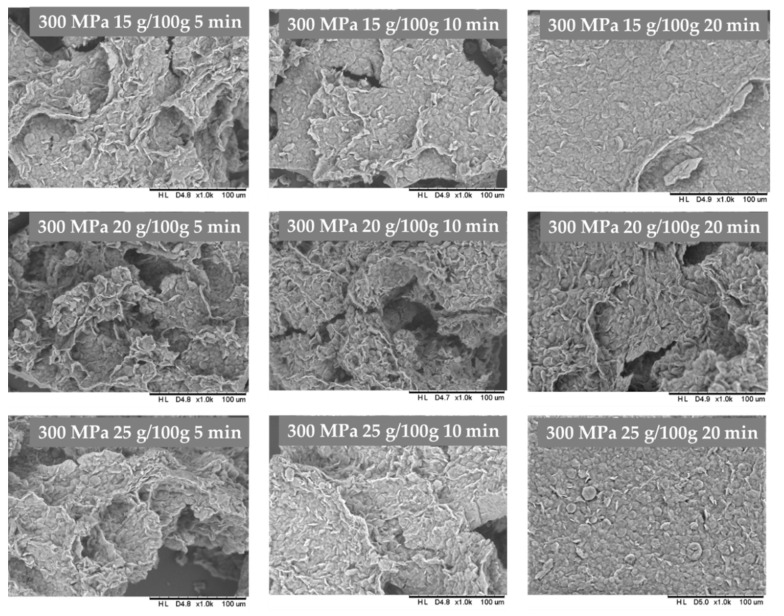
Influence of HHP time treatment under 300 MPa on inulin gel structure.

The inulin hydrogels structure was investigated basing on the images made with the scanning electron microscopy (Figure 5 and Figure 6). As it was improved in previous researches, pressure treatment changes morphologically hydrogels structure, by disorganization its original granular structure. After HHP with 500 MPa treatment the microstructure of gels is compressed, aggregated and with larger areas of disordered structures [24]. A similar effect was found during analyzing scanning electron microscopy images of hydrogels induced by 300 MPa pressure level, whereas gels induced by 150 MPa were packed loosely with no visible melting on the surface of granules, and less compressed structure, as well as visibly less smooth. It was also found that for higher inulin concentration (20–25 g/100 g) the time of HHP treatment also had influence on the hydrogel structure. The structure of hydrogels obtained with longer (20 min) HHP treatment time was almost flat as the granulated structure was almost invisible.

### 3.3. The Influence of HHP Level and Time Treatment on Yield Stress and Textural Properties of Inulin Hydrogels

**Figure 7:**
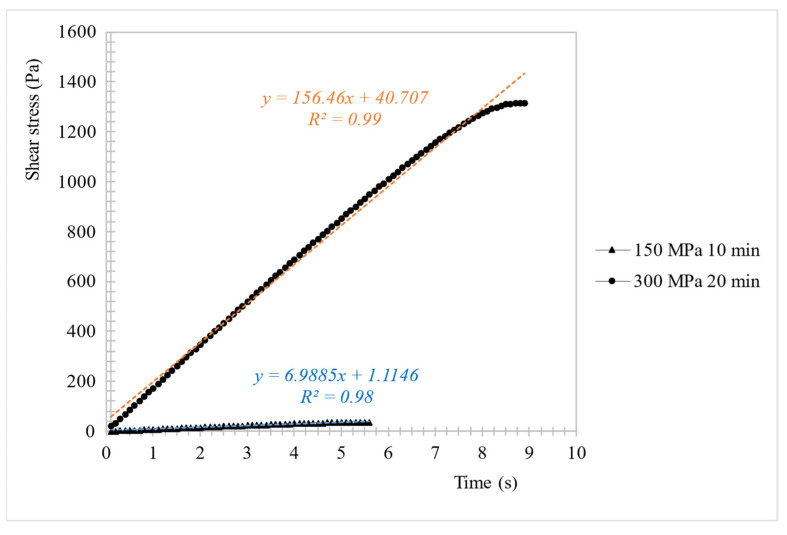
Sample shear stress curve measurements at constant share rate for two extreme measurement results: 150 MPa/10 min-20 g/100 g inulin concentration and 300 MPa/20 min-25 g/100 g concentration.

In order to determine the pressurization parameters on the structure of hydrogels, yield stress was determined. Yield stress is an initial resistance to flow under stress. Below the yield stress the sample will deform elastically, above the yield stress the sample will flow. It was found that the pressure had influence on gels resistance to stress (Table 1). Shear stress curve measurements at constant shear rate for two extreme measurement results: 150 MPa/10 min-20 g/100 g inulin concentration and 300 MPa/20 min-25 g/100 g concentration are presented in Figure 7. Generally, using lower pressure (150 MPa) made the gels more delicate (some of them were even semi-solid), characterized by much lower yield stress values than gels obtained at 300 MPa. These trends were found at each tested concentration of inulin, but at a lower analyzed concentration of inulin (15 g/100 g), higher pressure values changed their characteristic from liquid to solid regime. Similar changes were found also in the literature for glucomannan gels [19] or starch [20]. It was also found that at 300 MPa extending the pressurization time from 5 to 20 min caused gels to have greater resistance to the flow. Other authors also noticed the increase of consistency of tested polysaccharide products, after HHP treatment, what is more the increase depended on the pressure level as well as on time treatment [37,38]. This was probably due to the fact that higher pressure caused that the gel structure was more compressed and showed intact or partly disintegrated granular appearance. The changes are also confirmed by the microstructure analysis, and are in accordance to other authors’ researches in which polysaccharides such as starch [39] and cellulose [40] were suspended to HHP treatment.

The yield stress is also related to spreadability, as when the yield stress is achieved the gel displays irrecoverable deformation. Spreadability is described as a subjective, textural term and very often is defined as a stress required to create a uniform distribution over a surface [41]. It was found that, as in the case of yield stress, the pressure parameters affected the spreadability. Regardless of inulin concentration, the obtained gels were less spreadable (higher values of spreadability) after the inulin sols were subjected to higher values of pressure (Table 1). Moreover, it was found that at a concentration of 25 g/100 g of inulin, the pressurization time had a clear effect on the spreadability, the longer the pressurization time, the less spreadable the obtained gels were. At a lower concentration of inulin (20 g/100 g), this effect was observed only at a pressure of 300 MPa, while at a concentration of 15 g/100 g, significant changes caused only the extension of the pressure time (at a pressure of 300 MPa) from 5 to 20 min. This phenomenon might be due to the structural arrangement and intramolecular interactions of inulin as it is in case of other polysaccharides e.g., *Alyssum homolocarpum* seed gum [42].

When analyzing the influence of pressure parameters on firmness, it was found that exposure of sols to a higher pressure of 300 MPa as compared to 150 MPa resulted in the formation of gels with a stronger structure and higher firmness parameters (Table 1). This effect was observed regardless of the inulin concentration. At the same time, at the pressure of 300 MPa and higher inulin concentrations of 20 and 25 g/100 g, a tendency was found to strengthen the structure of the obtained gels along with the extension of the pressure time. Similar trends as in the case of the firmness regarding the pressure effect and its operating time were found for adhesiveness. Adhesiveness is a feature that is defined as the work essential to remove the probe from the gel sample, and in the food products it is very important to characterize its adherence to a spoon or tongue [43]. Regardless of the inulin concentration, exposure of the sols to higher pressure resulted in the formation of gels with higher adhesiveness. At the same time, for gels produced at a pressure of 300 MPa and a higher inulin concentration (>20 g/100 g), it was found that the extension of the pressurization time resulted in the formation of gels with higher adhesiveness. Based on literature data on the physical principles of HHP [44] it can be hypothesized that in high-pressure-induced inulin gels [24], as in other polysaccharides such as starch [45,46], the physical non-covalent interactions increased and became stronger with longer processing times.

### 3.4. The Influence of HHP Level and Time Treatment on Colour Parameters of Inulin Hydrogels

Some authors have reported that final color of polysaccharide gels’ samples is highly dependent on the operation pressure [19,47]. However analyzing the influence of pressure parameters on the color of the induced inulin gels, it was found that the pressure level (150–300 MPa) had no significant effect on the lightness of the color of the gels (L* color parameter values) (Table 2). Most of the gels obtained as a result of the higher pressure treatment were characterized by slightly lower average values of the color parameters a* and b*, although these differences were not always statistically significant. In order to comprehensively determine the influence of the pressure parameters on the gels’ color, the total color difference parameter (ΔE) was also determined. It was found that in most cases the ΔE values determined between the gels obtained after the 150 and 300 MPa pressure treatment were in the range of 1.0–2.0 (Table 3), which means that the only experienced observer could notice the differences in colors between such gels. There was also found a slight tendency to weaken the influence of the applied pressure on the color of the gels with increasing inulin concentration.

Regarding the effect of the pressure time (5–20 min) on the color of the gels, no clear influence of this pressure treatment parameter on the any of the determined parameters of the color of gels was found (Table 2). Analyzing the total color difference parameter, it was found that the pressure duration had a greater effect on the color of the gels formed after the treatment with lower than with higher pressure. This is indicated by higher values of the ΔE parameter of gels obtained after submitting the sols to a pressure of 150 MPa than 300 MPa (Table 3). 

At the same time, a slight tendency was observed to weakening the effect of the pressure time on the color of gels with increasing inulin concentration in the sol. According to literature data, even when higher pressure levels are applied for inulin gels’ induction there is observed no influence of the pressurization on L* color parameter [24]. However according to other studies some polysaccharides gels such as starch [48], glucomannan [19] or Jerusalem artichoke extracts [49] might darker after HHP treatment.

## 4. Conclusions

High-pressure treatment, even with relatively low parameters as for this technique (i.e., 150 MPa/5 min of time treatment), induces gelation of inulin and allows obtaining gel structures even at a lower concentration of inulin than required for other induction methods used in food industry. This gives the potential for wider use of HHP to also create inulin gel structures in products with a lower inulin content (i.e., 15 g/100 g) and where the use of higher pressures and longer time treatment is inadvisable. At the same time, by selecting the pressure parameters, it is possible to modify the characteristics of the created hydrogels and obtain gels with the required characteristics in a specific food product. The use of higher pressure (i.e., 300 MPa) allows us to increase the stability of the created gels and change their structure to more compressed, which results in higher yield stress, lower spreadability, harder and more adhesive gels. Extending the time treatment of HHP increases this effect, but mainly when higher pressure and a higher concentration of inulin are being used.

## Figures and Tables

**Table 1:** Physical properties of inulin gels obtained by different parameters of HHP induction.

Inulin Concentration	HHP Parameters	VGI [%]	Yield Stress [Pa]	Spreadability [N*s]	Firmness [N]	Adhesiveness [N*s]
15 g/100 g	150 MPa/5 min	100 ^aA^	0 ^aA^ ± 0	0.1 ^aA^ ± 0.0	0.0 ^aA^ ± 0.0	0.0 ^bC^ ± 0.0
	150 MPa/10 min	100 ^aA^	0 ^aA^ ± 0	0.1 ^aA^ ± 0.0	0.1 ^aA^ ± 0.0	0.0 ^bC^ ± 0.0
	150 MPa/20 min	100 ^aA^	0 ^aA^ ± 0	0.2 ^aA^ ± 0.0	0.1 ^aA^ ± 0.0	0.0 ^bC^ ± 0.0
	300 MPa/5 min	100 ^aA^	367 ^bA^ ± 24	2.4 ^bA^ ± 0.0	0.8 ^bA^ ± 0.1	−0.3 ^aC^ ± 0.1
	300 MPa/10 min	100 ^aA^	434 ^cA^ ± 12	2.4 ^bA^ ± 0.1	0.9 ^bA^ ± 0.3	−0.3 ^aC^ ± 0.1
	300 MPa/20 min	100 ^aA^	598 ^dA^ ± 10	2.7 ^cA^ ± 0.1	1.0 ^bA^ ± 0.1	−0.3 ^aC^ ± 0.0
20 g/100 g	150 MPa/5 min	100 ^aA^	0 ^aA^ ± 0	0.6 ^aB^ ± 0.0	0.1 ^aA^ ± 0.0	−0.1 ^cB^ ± 0.0
	150 MPa/10 min	100 ^aA^	38 ^bB^ ± 1	0.6 ^aB^ ± 0.0	0.1 ^aAB^ ± 0.0	−0.1 ^cB^ ± 0.0
	150 MPa/20 min	100 ^aA^	28 ^bB^ ± 1	0.6 ^aB^ ± 0.0	0.1 ^aA^ ± 0.0	−0.1 ^cB^ ± 0.0
	300 MPa/5 min	100 ^aA^	759 ^dB^ ± 4	4.2 ^bB^ ± 0.1	1.2 ^bB^ ± 0.0	−0.6 ^bB^ ± 0.0
	300 MPa/10 min	100 ^aA^	712 ^cB^ ± 7	4.6 ^cB^ ± 0.0	1.5 ^cB^ ± 0.0	−0.6 ^aB^ ± 0.0
	300 MPa/20 min	100 ^aA^	875 ^eB^ ± 5	4.8 ^dB^ ± 0.0	1.5 ^cA^ ± 0.0	−0.7 ^aB^ ± 0.0
25 g/100 g	150 MPa/5 min	100 ^aA^	41 ^aB^ ± 4	1.2 ^aC^ ± 0.1	0.3 ^aB^ ± 0.1	−0.1 ^dA^ ± 0.0
	150 MPa/10 min	100 ^aA^	68 ^aC^ ± 3	1.5 ^bC^ ± 0.1	0.2 ^aB^ ± 0.1	−0.1 ^dA^ ± 0.0
	150 MPa/20 min	100 ^aA^	65 ^aC^ ± 2	1.6 ^cC^ ± 0.0	0.2 ^aB^ ± 0.0	−0.1 ^dA^ ± 0.0
	300 MPa/5 min	100 ^aA^	775 ^bB^ ± 7	6.3 ^dC^ ± 0.1	1.9 ^bC^ ± 0.1	−0.9 ^cA^ ± 0.0
	300 MPa/10 min	100 ^aA^	932 ^cC^ ± 4	8.0 ^eC^ ± 0.0	2.1 ^bC^ ± 0.1	−1.0 ^bA^ ± 0.0
	300 MPa/20 min	100 ^aA^	1274 ^dC^ ± 50	8.4 ^fC^ ± 0.0	3.0 ^cB^ ± 0.6	−1.3 ^aA^ ± 0.0

Values are mean ± SD (*n* = 3). Values followed by the same lower or uppercase letters do not differ significantly according to Tukey’s test (*p* < 0.05). Lowercase letters (a–f) indicate the effect of pressure parameters at a given concentration of inulin; uppercase letters (A–C) indicate the effect of inulin concentration at given pressure parameters.

**Table 2:** Color parameters of inulin gels obtained by different parameters of HHP induction.

Inulin Concentration	HHP Parameters	L*	a*	b*
15 g/100 g	150 MPa/5 min	85.8 ^bA^ ± 1.5	−1.5 ^bA^ ± 0.2	0.9 ^cdA^ ± 0.0
	150 MPa/10 min	84.0 ^abA^ ± 0.1	−1.6 ^bA^ ± 0.1	1.1 ^dA^ ± 0.0
	150 MPa/20 min	83.3 ^aA^ ± 1.0	−1.7 ^abA^ ± 0.1	0.7 ^cA^ ± 0.1
	300 MPa/5 min	85.3 ^abA^ ± 1.1	−1.8 ^abA^ ± 0.1	0.4 ^bA^ ± 0.0
	300 MPa/10 min	85.8 ^bA^ ± 0.2	−1.9 ^aA^ ± 0.0	0.0 ^aA^ ± 0.0
	300 MPa/20 min	85.1 ^abA^ ± 0.4	−1.8 ^abA^ ± 0.1	0.0 ^aA^ ± 0.2
20 g/100 g	150 MPa/5 min	87.5 ^abA^ ± 1.5	−1.4 ^dA^ ± 0.0	0.9 ^bA^ ± 0.0
	150 MPa/10 min	87.1 ^abB^ ± 0.2	−1.5 ^bcA^ ± 0.0	1.6 ^dB^ ± 0.1
	150 MPa/20 min	85.5 ^aA^ ± 1.5	−1.6 ^bB^ ± 0.0	1.2 ^cB^ ± 0.1
	300 MPa/5 min	86.3 ^abAB^ ± 0.1	−1.7 ^aAB^ ± 0.0	0.3 ^aA^ ± 0.0
	300 MPa/10 min	88.13 ^bC^ ± 0.1	−1.4 ^cdC^ ± 0.0	0.9 ^bB^ ± 0.0
	300 MPa/20 min	86.3 ^abB^ ± 0.1	−1.7 ^aB^ ± 0.0	0.3 ^aB^ ± 0.0
25 g/100 g	150 MPa/5 min	87.8 ^aA^ ± 0.3	−1.4 ^cdA^ ± 0.0	2.7 ^bcB^ ± 0.1
	150 MPa/10 min	87.8 ^aB^ ± 1.5	−1.3 ^eB^ ± 0.0	3.1 ^dC^ ± 0.2
	150 MPa/20 min	88.4 ^aB^ ± 0.6	−1.4 ^cA^ ± 0.0	2.7 ^cC^ ± 0.1
	300 MPa/5 min	87.0 ^aB^ ± 0.0	−1.6 ^bB^ ± 0.0	2.3 ^abB^ ± 0.1
	300 MPa/10 min	86.8 ^aB^ ± 0.1	−1.8 ^aB^ ± 0.0	2.1 ^aC^ ± 0.1
	300 MPa/20 min	87.2 ^aC^ ± 0.1	−1.3 ^deC^ ± 0.0	2.3 ^aC^ ± 0.0

Values are mean ± SD (*n* = 3). Values followed by the same lower or uppercase letters do not differ significantly according to Tukey’s test (*p* < 0.05). Lowercase letters (a,b) indicate the effect of pressure parameters at a given concentration of inulin; uppercase letters (A,B) indicate the effect of inulin concentration at given pressure parameters.

**Table 3:** The color differences parameter (ΔE) between inulin hydrogels prepared with different HHP parameters.

Inulin Concentration and HHP Parameters			Inulin 25 g/100 g						Inulin 20 g/100 g						Inulin 15 g/100 g					
			300 MPa			150 MPa			300 MPa			150 MPa			300 MPa			150 MPa		
			20 min	10 min	5 min	20 min	10 min	5 min	20 min	10 min	5 min	20 min	10 min	5 min	20 min	10 min	5 min	20 min	10 min	5 min
inulin 15 g/100 g	150 MPa	5 min	2.11	1.81	2.08	3.19	3.41	2.75	1.36	2.29	1.32	0.96	1.54	1.64	2.00	1.68	1.73	2.61	1.91	-
		10 min	3.49	2.96	3.27	4.75	4.37	4.18	2.50	4.18	2.45	1.60	3.17	3.56	1.71	2.18	1.69	1.00	-	
		20 min	4.32	3.77	4.11	5.58	5.15	5.02	3.07	4.88	3.03	2.31	3.95	4.25	2.05	2.61	2.09	-		
	300 MPa	5 min	2.80	2.32	2.69	3.98	3.73	3.52	1.04	2.89	0.97	1.41	2.25	2.40	0.96	1.07	-			
		10 min	2.83	2.35	2.70	3.90	3.97	3.45	0.71	2.59	0.67	1.90	2.14	2.21	0.65	-				
		20 min	3.22	2.69	3.06	4.37	4.26	3.88	1.26	3.16	1.23	1.95	2.59	2.76	-					
inulin 20 g/100 g	150 MPa	5 min	1.80	1.86	1.92	2.16	3.19	2.13	1.64	1.31	1.63	2.07	1.37	-						
		10 min	0.78	0.68	0.80	1.79	2.11	1.33	1.51	1.25	1.53	1.81	-							
		20 min	2.20	1.82	2.12	3.35	3.02	2.90	1.61	2.62	1.55	-								
	300 MPa	5 min	2.24	1.82	2.16	3.28	3.36	2.86	0.10	1.97	-									
		10 min	1.68	1.85	1.85	1.95	2.57	1.81	1.93	-										
		20 min	2.22	1.82	2.15	3.26	3.34	2.84	-											
Inulin 25 g/100 g	150 MPa	5 min	0.74	1.30	0.94	0.59	1.40	-												
		10 min	1.57	1.86	1.65	1.54	-													
		20 min	1.28	1.84	1.50	-														
	300 MPa	5 min	0.39	0.41	-															
		10 min	0.71	-																
		20 min	-																	
Values are mean (*n* = 3).																				
0 < ΔE < 1		1 < ΔE < 2				2< ΔE < 3.5					3.5 < ΔE < 5					5 < ΔE				
observer does not notice the difference		only experienced observer can notice the difference				unexperienced observer also notices the difference					clear difference in color is noticed					observer notices two different colors				
